# The pathophysiology of mixed Alzheimer's disease and vascular dementia

**DOI:** 10.7150/thno.118737

**Published:** 2025-09-03

**Authors:** Mutaz Sarhan, Christian Wohlfeld, Ariel Perry-Mills, Jeffrey Meyers, James Fadel, E. Angela Murphy, Leonardo Bonilha, Daping Fan

**Affiliations:** 1 Department of Cell Biology and Anatomy, University of South Carolina School of Medicine, Columbia, SC, USA.; 2 Department of Physiology, Pharmacology, and Neuroscience, University of South Carolina School of Medicine, Columbia, SC, USA.; 3 Department of Neurology, University of South Carolina School of Medicine, Columbia, SC, USA.; 4 Department of Pathology, Microbiology, and Immunology, University of South Carolina School of Medicine, Columbia, SC, USA.

**Keywords:** Alzheimer's disease, vascular dementia, mixed dementia, pathophysiology, experimental models

## Abstract

Mixed dementia is caused most often by the coexistence of Alzheimer's disease (AD) and vascular dementia (VaD) pathologies. This disease presents challenges due to its complex, dual pathology. In this review, we summarize the current understanding of the pathophysiology of AD and VaD, with a particular emphasis on vascular factors that accelerate or exacerbate AD pathology. We then describe animal models, in vitro cell culture systems, and brain organoid models that have been developed or are currently being developed to elucidate the neurodegenerative and vascular components of MD. This review provides an evaluation of the panorama of factors that influence MD pathophysiology and how basic science models can incorporate these factors to advance the knowledge related to the mechanisms of the disease and guide the screening of novel therapeutic approaches.

## 1. Introduction

Mixed dementia (MD) occurs when two or more distinct pathologies contribute to cognitive decline in the same individual. MD most commonly results from the co-existence of Alzheimer's disease (AD) and vascular dementia (VaD). For clarity, we hereafter refer to MD as the combination of AD and VaD in this review. Despite its clinical importance, evaluating MD is challenging due to the substantial overlap in symptoms between AD and VaD, making it difficult to determine which pathology is primarily responsible for neurodegeneration and cognitive decline in affected individuals 1. The complexity in the pathophysiology of MD can lead to a lack of standardized diagnostic criteria to distinguish the influence of AD vs. VaD in MD 2.

An understanding of the pathophysiological interplay between vascular disease and neurodegeneration is essential for improving the evaluation of MD. Vascular incidents can directly lead to the loss of brain cells 3, but ischemic events and reduced cerebral blood flow can also trigger increased amyloid β (Aβ) deposition and tau phosphorylation 4.

Despite its clinical significance, MD remains under-studied compared to the extensive studies focused on AD and VaD. In this review, we summarize the current understanding of the pathophysiology of AD, VaD, and MD, with a focus on the vascular factors that may accelerate or worsen AD pathology. We also examine emerging experimental platforms that are contributing to bridging the knowledge gaps in MD research, highlighting their potential to unravel disease mechanisms and support the development of targeted interventions.

## 2. Alzheimer's disease

AD is the most common form of dementia and accounts for 60-80% of dementia cases 5. AD currently affects an estimated 45 million people worldwide, and its prevalence is expected to triple by the year 2050 6. These figures are likely underestimated due to cases of misdiagnosis or undiagnosed individuals as results of socioeconomic barriers and/or provider knowledge gaps and misperceptions 7-9. Because normal aging is associated with cognitive decline, distinguishing age-related changes from AD can also be challenging 10.

**Figure 1:**
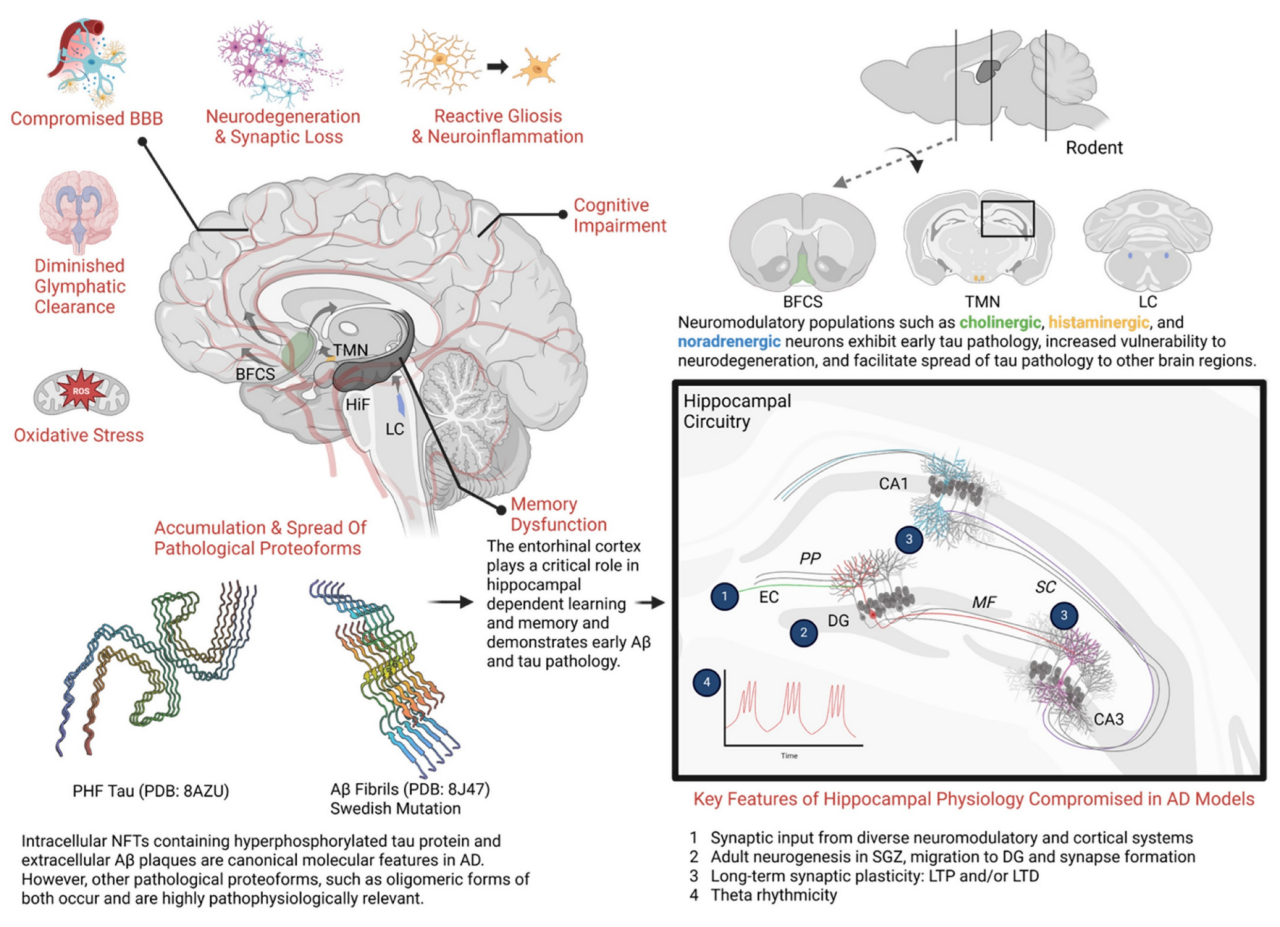
** General pathophysiologic features across the progression of Alzheimer's disease.** Schematic overview of key selected pathophysiologic features present in AD. Genetics, environmental factors, aging, and the presence of other disease states (diabetes, stroke, traumatic brain injury, etc.) strongly influence these key factors in clinical populations and animal models. (Bottom left) Accumulation and propagation of pathological proteoforms of tau protein and Aβ peptides has been a long-appreciated mechanism underlying AD. Initial histological studies in human cadavers revealed neurofibrillary tangles (NFTs) and extracellular β-amyloid plaques as characteristic features of AD 203. However, advances in structural biology, proteomics, neuroimaging, and the use of transgenic animal models have provided a richer perspective of these two central molecular features. Shown here, smaller oligomeric forms such as paired helical filaments (PHF) of tau and Aβ fibrils isolated from AD cadavers, available on Protein Data Bank. (Top right) While the vast majority of neuronal populations are excitatory or inhibitory, a smaller minority of neurons comprising neuromodulatory systems appear highly relevant to early AD pathogenesis. Shown here, cholinergic neurons in the basal forebrain, also known as the basal forebrain cholinergic system (BFCS), histaminergic neurons whose cell bodies reside in the tuberomammillary nucleus (TMN) in the hypothalamus, and noradrenergic neurons in the locus coeruleus (LC). These neuromodulatory populations send projections to the hippocampal formation and broadly innervate cortical regions, where they influence cognition and memory. Importantly, these neurons are unmyelinated, have large projection fields and extensive metabolic requirements, which may make them uniquely susceptible to oxidative stress and neurodegeneration. Additionally, evidence suggests these brain regions, especially the LC, provide a neuroanatomical site for the early formation, accumulation, and eventual spread of tau pathology 204, 205. Characteristically, tau pathology has been observed early in the LC prior to spread to the entorhinal cortex (EC), hippocampus, and neocortex. Whereas early Aβ pathology is often observed in the EC prior to detection in other brain regions. (Bottom Right) The trisynaptic circuit in the hippocampus is essential for encoding, retrieval and other aspects of memory. Projections from the EC to the dentate gyrus (DG) form the perforant pathway (PP). Subsequently, DG neurons project to CA3 via mossy fiber pathway (MF)—which in turn, sends projections to CA1 via the Schaffer collateral pathway (SC). Unique cell types (e.g. place cells, grid cells, etc.) occupy regions of the hippocampal formation (HiF) and functionally support aspects of memory 206. Synaptic plasticity, such as long-term potentiation and long-term depression and neural oscillations such as theta rhythmicity represent critical neural mechanisms across the trisynaptic circuit that support memory and are compromised in various animal models of AD 207. Adult neurogenesis represents a rather unique mechanism occurring in subgranular zone (SGZ). Nascent adult neurons from the SGZ migrate to granular layer in the DG and send projection to CA3. This effect supports memory processes and is compromised in AD models 208 . Each of these hippocampal neural mechanisms is also supported by an array of neuromodulatory inputs, like the BFCS. Taken together, these key selected pathophysiologic factors facilitate synaptic dysfunction and neurodegeneration of the hippocampal formation which corresponds with the emergence of memory dysfunction in animal models. (Unshown) In late stages of AD, these hallmark pathological features extend broadly through the neocortex, which is accompanied by significant atrophy, synaptic loss, ventricular dilation and profound cognitive impairment as seen in patients and animal models 209, 210. *Molecular structures are available via PDB:* The Protein Data Bank H.M. Berman, J. Westbrook, Z. Feng, G. Gilliland, T.N. Bhat, H. Weissig, I.N. Shindyalov, P.E. Bourne (2000) *Nucleic Acids Research*, **28**: 235-242. *Created in BioRender. Wohlfeld, C. (2025) https://BioRender.com/x43y595*.

The pathogenesis of AD is complex, multi-faceted, and not entirely understood 11. Hallmark features of AD pathology include (I) age-dependent neurodegeneration that often manifests as gross neuroanatomical changes 12-14, (II) the accumulation of pathological protein aggregates such as Aβ plaques and neurofibrillary tangles (NFTs) 12, (III) proliferation of glia (gliosis) and alterations in glial function that disrupt neuroimmune function and the blood brain barrier 12, 15, and (IV) an altered cellular landscape featuring multifaceted changes in cell signaling and metabolism, excessive oxidative stress, dysfunctional autophagy, impaired cell transport, and synaptic dysfunction 16-19. Key pathophysiologic aspects of AD are illustrated in **Figure 1**.

### 2.1. Aβ plaques

Amyloid precursor protein (APP) is a transmembrane protein that is believed to be involved in processes like synaptic formation, neuronal growth, and cell adhesion. APP is normally processed through the non-amyloidogenic pathway. This pathway begins with APP being cleaved by α-secretase. This cleavage prevents the formation of Aβ peptides by producing a large soluble fragment, sAPPα, and a smaller membrane-bound fragment, C-Terminus Fragment α (CTFα). γ-secretase then cleaves CTFα to produce a non-toxic peptide called p3 and the APP intracellular domain (AICD) 20. In AD, however, the cleavage of APP is processed through the amyloidogenic pathway in which APP is cleaved by β-secretase (BACE1) producing a fragment called C-Terminus Fragment β (CTFβ). γ-secretase then cleaves CTFβ resulting in the formation of Aβ peptides that are released into the extracellular space. The two major Aβ peptides produced are Aβ40 and Aβ42. While Aβ40 is the most common form, Aβ42 is more prone to aggregation and is primarily found in amyloid plaques 21, 22. Presenilin 1 (PSEN1) and presenilin 2 (PSEN2) are the two most important integral components of the γ-secretase complex. Changes in the functions of α-, β-, and γ-secretases are linked to early-onset familial AD 23, 24.

The accumulation of Aβ is also regulated through degradation and clearance mechanisms. The two main enzymes involved in degrading Aβ are neprilysin (NEP) and insulin-degrading enzyme (IDE). NEP degrades extracellular Aβ, but its levels decrease with age, contributing to age-related increased risk of AD. IDE exhibits a higher affinity for insulin than Aβ. Reduced IDE activity has been linked to AD, especially in individuals with type II diabetes 25, 26, underscoring the interplay between insulin regulation and Aβ metabolism.

In addition to degradation, Aβ can be cleared from the brain through the blood-brain barrier (BBB). The low-density lipoprotein receptor-related protein (LRP) helps transport Aβ out of the brain. Disruption in the clearance process could lead to the buildup of Aβ and the formation of Aβ plaques in the brain 27, 28. The accumulation of Aβ due to increased production, reduced degradation, or impaired clearance are central features of AD pathology; therefore, therapeutic strategies are focused on inhibiting β-secretase and γ-secretase and enhancing the degradation or clearance of Aβ 29, 30.

### 2.2. Tau pathology

Tau is a microtubule-associated protein primarily found in neurons. Its main function is stabilizing microtubules, which are dynamic structures that provide support to axons and dendrites, thereby allowing for neuronal processing to remain intact and functional 31. In a healthy brain, tau undergoes a cycle of phosphorylation and dephosphorylation. Phosphorylation of tau causes it to detach from microtubules, enabling microtubule restructuring and reorganization. Tau is then dephosphorylated and reattaches to the microtubules to stabilize the newly organized structure. This cycle maintains the neuronal structure and facilitates intracellular transport 31.

In AD, tau undergoes abnormal hyperphosphorylation that disrupts the normal cycle of microtubule organization. Hyperphosphorylation of tau reduces its affinity to the microtubules, causing them to destabilize and disassemble. Instead of binding to microtubules, hyperphosphorylated tau aggregates to form insoluble fibrils, which accumulate within neurons forming NFTs, one of the defining pathological features of AD and commonly associated with neurodegeneration in general 32. Studies have shown that NFTs can propagate from one neuron to another, spreading in a prion-like manner. This spreading mechanism may explain the progressive nature of AD, as tau pathology spreads from initially affected regions (so-called “seed” regions) to other parts of the brain 33.

### 2.3. Microglial activation

In a healthy brain, microglia constantly survey and monitor the brain's microenvironment. One of the primary roles of microglia is to phagocytose and clear debris and dead cells. This process is essential for preventing the accumulation of toxic materials that could harm neurons and impair their function 34. The clearance of debris by microglia is facilitated by the glymphatic system. This system transports the waste collected by microglia from the brain's interstitial space to the cerebrospinal fluid (CSF), which is then directed to meningeal lymphatic vessels. These vessels drain the waste into peripheral lymph nodes, then to the bloodstream, and the waste is finally cleared by the liver and kidneys 35, 36. The glymphatic system is more active during sleep, making restful sleep crucial for the efficient clearance of waste products from the brain 36. Microglia also play a significant role in ensuring the proper formation of neuronal circuits, a process called synaptic pruning, by removing excess synapses. This process is imperative for cognitive function as it allows for efficient processing of information and maintenance of brain plasticity. Microglia also regulate inflammation by producing anti-inflammatory cytokines and maintaining a balance between pro- and anti-inflammatory signals in the brain 37.

In AD pathogenesis, microglia can become overactivated, leading to a continuous inflammatory response. The accumulation of Aβ plaques and NFTs causes microglia to shift from a homeostatic to an activated state, characterized by the release of pro-inflammatory cytokines, chemokines, and reactive oxygen species (ROS) 38. While the initial activation is a protective response to clear Aβ plaques and other debris, chronic activation of microglia can lead to a state of chronic inflammation. This can lead to impairment of the glymphatic system, resulting in reduced clearance and increased aggregation of Aβ and tau, exacerbating AD pathology 39, 40. In addition, the prolonged release of inflammatory mediators by activated microglia disrupts the integrity of the BBB, compromising the 'immune privilege' by allowing peripheral immune cells to enter the brain and amplify the inflammatory response. This vicious cycle of chronic inflammation is a key driver in the progression of AD 41.

### 2.4. Genetic mutations in AD

The majority of AD cases are sporadic, primarily caused by environmental and lifestyle factors, while only a small percentage are attributed to known hereditary causes 42. Familial AD (FAD), typically associated with early-onset AD, only counts for about 1-5% of all AD cases. The primary genetic mutations involved in FAD include those in the APP, PSEN1, and PSEN2 genes. These mutations lead to abnormal production or accumulation of Aβ plaques that triggers AD pathology earlier in life 43. In addition to these mutations, the *APOE* ε4 allele is the most studied genetic risk factor associated with late-onset AD (LOAD). Individuals with just one copy of the *APOE* ε4 allele have a 3-4 times higher likelihood of developing AD relative to those with two copies of *APOE* ε3, the most common form, while those with two copies of the *APOE* ε4 allele face up to a 12-fold increased risk of developing AD 44. Conversely, *APOE* ε2 may be protective against AD. In addition, mutations in several other genes - such as Trem2, clusterin, PICALM, CR1, BIN1, ABCA7, and CD33 - have been linked to an increased risk for LOAD, although their associations with AD risk are significantly weaker compared to that of the APOE ε4 allele.

### 2.5. Vascular contributions to AD pathology

While Aβ and tau pathologies are the key features of AD, vascular components also contribute to disease progression. For instance, impaired cerebral blood flow can accelerate the progression of AD; a reduction in cerebral blood flow deprives the brain of oxygen and other essential nutrients needed for normal brain functionality. Further, disruptions in the BBB can result in harmful substances entering the brain, leading to inflammation and neurotoxicity. Moreover, amyloid angiopathy weakens the vascular integrity of the blood vessels, increasing the likelihood of bleeding and vascular damage in the brain. These vascular abnormalities interplay with core AD pathologies, contributing to and accelerating AD development 45.

#### 2.5.1. Reduced cerebral blood flow

One significant vascular abnormality that contributes to AD pathology is reduced cerebral blood flow, or chronic cerebral hypoperfusion, which deprives neurons of oxygen and essential nutrients, ultimately leading to neuronal death 46. Neurons are especially susceptible to hypoxic conditions due to their high energy demand and low glutathione concentrations, which limits their ability to combat oxidative stress 47, 48. Considering that oxidative stress is a central pathological mechanistic feature in AD, hypoxia and oxidative stress serve as a mechanistic common ground of AD and VaD.

In response to hypoxia, neural tissue enacts transcriptional regulation of genes implicated in angiogenesis 49. As an example, hypoxia inducible factors (HIFs) are transcription factors that are responsive to ROS and stabilized under hypoxia 49. HIFs bind DNA at hypoxia response elements (HREs) and upregulate the expression of genes relevant to the VEGF pathway 49. However, it has also been shown that HIFs may mediate maladaptive transcriptional regulation of BACE and γ-secretase 49. Given the pathological role of BACE and γ-secretase in APP processing that favors increased Aβ_42_ production, HIF-mediated transcriptional regulation under hypoxic conditions may further advance Aβ pathology. In addition, chronic hypoperfusion exacerbates Aβ aggregation due to impaired Aβ clearance, and impairs the glymphatic system in clearing these toxic Aβ aggregates 28.

Reduced cerebral blood flow has also been shown to increase the hyperphosphorylation of the tau protein. Hypoxic conditions in the brain can activate kinases that modify the tau protein, lead to increased hyperphosphorylation and aggregation of neurofibrillary tangles, and thus contribute to synaptic dysfunction and neuronal death in AD 46.

#### 2.5.2. Blood-brain barrier disruption

The BBB is a highly specialized neurovascular structure critical for maintaining cerebral homeostasis. Its dysfunction is increasingly recognized as a central driver of AD pathogenesis, particularly in patients with vascular comorbidities. Vascular insults—such as hypertension, diabetes, and atherosclerosis—induce endothelial activation, pericyte degeneration, and degradation of tight junction proteins, compromising BBB integrity 50, 51. Patients with vascular damage are more likely to have a compromised BBB, permitting neurotoxic substances such as plasma proteins, inflammatory mediators, and other harmful molecules to leak into the brain parenchyma 51. This can lead to a cascade of uncontrolled inflammatory responses driven by activated microglia 50. Over time, the uncontrolled microglial response further compromises the BBB, facilitating the infiltration of immune cells into the brain and promoting the release pro-inflammatory cytokines and oxidative molecules. Astrocytes also respond by adopting a pro-inflammatory phenotype, releasing complement proteins that exacerbate synaptic pruning and neuronal loss 52. This perpetuates a vicious cycle that accelerates neurodegeneration in patients 38.

BBB disruption also impairs Aβ clearance through downregulation of LRP1 at the endothelial membrane and decreases the ability of the glymphatic system to facilitates the drainage of Aβ during sleep 39, 53. This is further compounded by matrix metalloproteinase-9 (MMP-9) overexpression, which is induced by oxidative stress. MMP9 degrades the basal lamina collagen, increases BBB permeability, and facilitates leukocyte infiltration 54.

#### 2.5.3. Cerebral amyloid angiopathy

Another vascular component of AD is the development of cerebral amyloid angiopathy (CAA). In CAA, Aβ deposits accumulate within the walls of cerebral arteries, progressively reducing blood flow in the affected areas 55. This is due to the impaired intramural periarterial drainage (IPAD), where aging-associated basement membrane thickening and APOE ε4-driven fibrillogenesis obstruct perivascular Aβ clearance 55, 56. CAA can further lead to vascular occlusions and even ruptures, result in microhemorrhages and microinfarcts, and thus more severely disrupt blood flow to certain regions of the brain, exacerbating neurodegeneration and cognitive decline 57. Neuropathological studies have shown that CAA-associated microinfarcts colocalize with phosphorylated tau in the neocortex, accelerating Tau tangle formation through activation of glycogen synthase kinase-3β (GSK-3β) 58. Additionally, cerebral microbleeds (CMBs) increase the risk of dementia and are correlated with executive dysfunction, which is a common symptom in both VaD and MD 59.

## 3. Vascular dementia (VaD)

VaD is the second most common form of dementia, accounting for 15-20% of all dementia cases worldwide 60. It is caused by a reduction or loss of blood flow to the brain, which result in deprivation of oxygen and essential nutrients, leading to neuronal death and brain lesions, and eventually impairments in cognitive function. Common causes for VaD are stroke, cerebral small vessel disease, and cerebral hypoperfusion 61, often resulting from conditions such as hypertension, hyperlipidemia, and diabetes and insulin resistance. As discussed in the previous section, these vascular pathologies may accelerate AD development, but they can also independently cause VaD in the absence of AD pathology. Hypertension, hyperlipidemia, and diabetes and insulin resistance not only cause vascular pathologies and VaD, but also directly or indirectly contribute to AD development; therefore, we will discuss these conditions later in the MD section.

### 3.1. Ischemic or hemorrhagic stroke

Ischemic stroke, caused by obstructed blood flow, can lead to necrosis and gliosis and damage regions and networks that are critical for cognitive processing. Over time, the accumulation of multiple ischemic events can lead to a progressive decline in cognitive abilities. This gradual accumulation of damage, particularly in the form of small, deep brain infarcts known as lacunar infarcts, is a common pathway through which ischemic strokes lead to VaD 62.

Hemorrhagic strokes, caused by the rupture of a blood vessel, are less common than ischemic strokes, but also play a significant role in the development of VaD. The presence of blood within the brain tissue can lead to a cascade of detrimental events, including inflammation, oxidative stress, and excitotoxicity, all of which exacerbate neuronal injury and contribute to cognitive decline 63. In some cases, increased intracranial pressure can also compress nearby brain structures, causing a secondary ischemic stroke that perpetuates further injury. The location of the hemorrhage is crucial in determining the specific cognitive deficits. For example, a hemorrhage in the basal ganglia or thalamus can lead to significant impairments in attention and executive function 64.

The cumulative effect of stroke on the brain, either through ischemic or hemorrhagic mechanisms, is a significant contributor to the development and progression of VaD. The damage from these strokes not only causes immediate cognitive impairments but also increases vulnerability to further decline as subsequent vascular events may follow.

### 3.2. Cerebral small vessel disease

Cerebral small vessel disease (SVD) is characterized by a variety of pathological changes, which impair the function of small blood vessels. One of the hallmarks of SVD is the thickening and hardening of the wall of small arteries, known as arteriolosclerosis. This process is often driven by hypertension, which increases mechanical stress on the vessel walls 46. This impairs the vessel's ability to maintain adequate cerebral blood flow and diminishes its responsiveness to fluctuations in blood pressure. White matter hyperintensities (WMHs) are characteristic of SVD and can be visualized using MRI. WMHs represent regions of demyelination of the axons due to the loss of local blood flow. The progressive accumulation of lesions in the white matter is associated with a decline in cognitive function, particularly in areas such as executive function, processing speed, and attention 46.

SVD also leads to the formation of lacunar infarcts, which are small, deep brain infarcts caused by the occlusion of a single penetrating artery. These infarcts typically occur in subcortical structures such as the basal ganglia, thalamus, and internal capsule, and can cause significant cognitive impairment 65. Unlike larger cortical strokes that cause abrupt changes in cognitive functioning, lacunar infarcts are more subtle and cause small neurological changes that can accumulate over time leading to progressive cognitive decline.

In addition, SVD may be associated with cerebral microbleeds, which are small, asymptomatic hemorrhages that occur in the brain. These microbleeds are typically caused by the rupture of small, fragile blood vessels, and are often due to hypertension 66. While cerebral microbleeds themselves do not usually cause immediate cognitive impairments, their presence indicates vascular damages and is associated with an increased risk of cognitive decline and dementia.

### 3.3. Cerebral hypoperfusion

In addition to abovementioned stroke that causes acute cerebral hypoperfusion, chronic cerebral hypoperfusion due to various systemic conditions is also common in VaD pathogenesis. These conditions include atherosclerosis, carotid artery stenosis/occlusion, chronic heart failure, and chronic hypotension, among others; they reduce the blood flow into the brain. The chronic and progressive nature of cerebral hypoperfusion leads to a gradual decline in cognitive function, resulting in VaD. The regions of the brain most vulnerable to hypoperfusion are the hippocampus, prefrontal cortex, and white matter tracts, all of which are critical for memory, executive function, and processing speed 60. As these regions become damaged, individuals experience symptoms such as memory loss, difficulty with problem-solving, slowed thinking, and impaired judgment. One of the key features of VaD caused by cerebral hypoperfusion is its insidious onset and gradual progression. This gradual cognitive decline is often accompanied by vascular risk factors such as hypertension, hyperlipidemia, and diabetes, all of which exacerbate hypoperfusion 67.

## 4. Mixed dementia

Mixed dementia is a disorder in which most often the pathologies of AD and VaD coexist and interact, resulting in a more complex clinical manifestation than either condition alone. The combined impact of neurodegeneration and vascular damage in MD leads to a variety of cognitive, motor, and behavioral symptoms that challenge diagnosis and management. Both AD and VaD present with prominent cognitive symptoms that include memory loss and cognitive decline, which can make a differential diagnosis of MD challenging 68.

On the one hand, MD often presents with subtle vascular changes that are easily overlooked in patients with obvious AD pathology; on the other hand, AD aspects are often ignored in MD patients with overwhelming post-stroke VaD pathology. As a result, clinicians may misattribute symptoms of MD to either pure AD or VaD, potentially leading to an underestimation of MD's true prevalence 69. Indeed, studies have shown that the incidence of MD ranges from 2% to 60% depending on the criteria used and the population studied 2. Neuropathological studies provide more accurate insights, as autopsy examinations are the gold standard for diagnosis and frequently reveal coexisting pathologies in patients who were diagnosed with either AD or VaD during their lifetime. For instance, Schneider *et al.* 70 conducted an autopsy-based study and found that 38% of elderly dementia patients exhibited signs of both AD and cerebrovascular disease, 30% had pure AD, and only 12% had pure VaD. These findings suggest that MD could be far more common than previously reported, particularly in patients over the age of 75 2. Autopsy studies in individuals with SVD have also shown concurrent Aβ plaques and NFTs characteristic of AD pathology 71. This suggests that many individuals diagnosed with either AD or VaD during their life may have actually been affected by MD. As the population ages, MD is becoming increasingly prevalent. More research is needed to better understand its pathogenesis and improve management outcome.

### 4.1. Pathophysiology of MD

**Figure 2:**
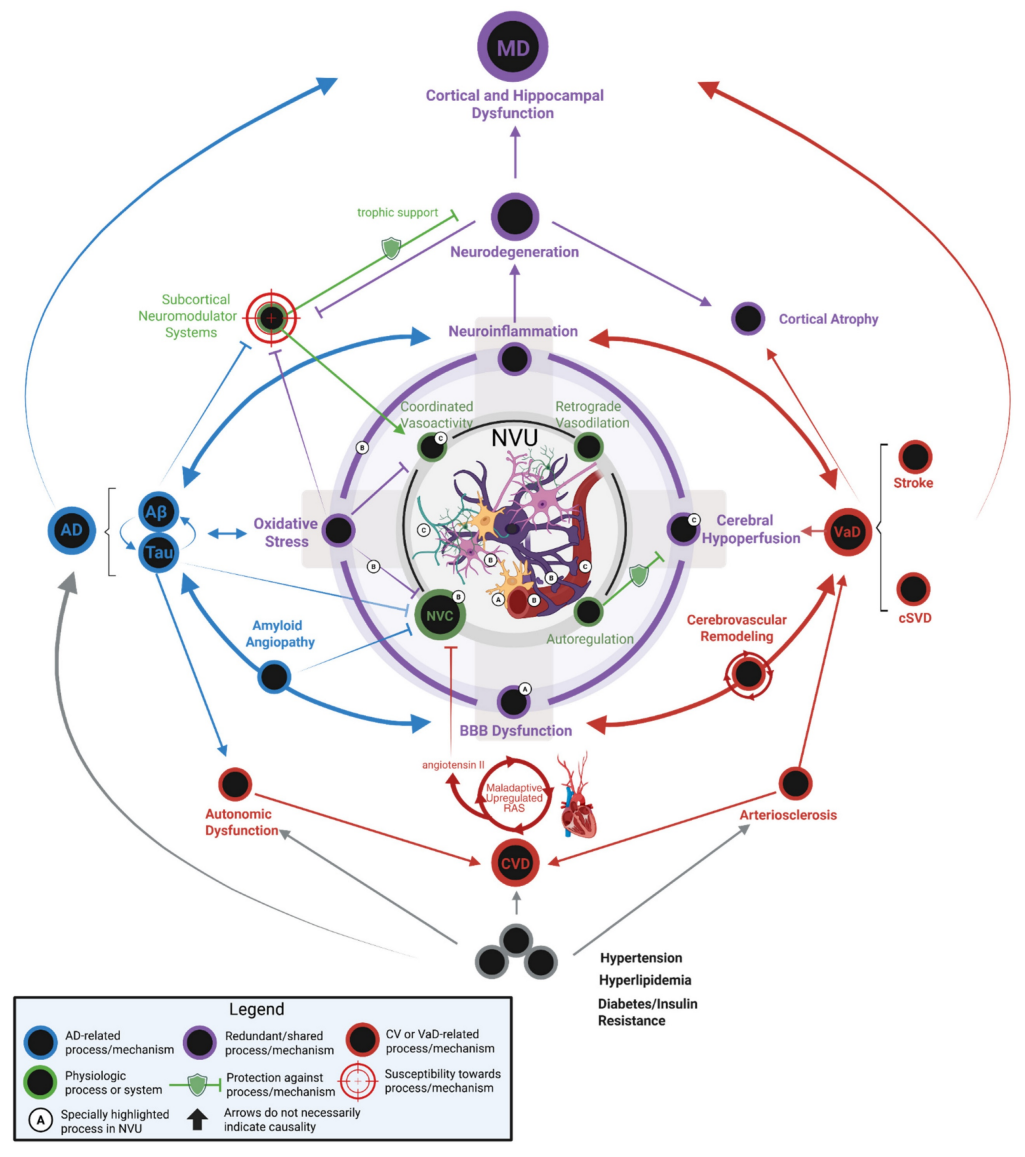
** Mixed dementia pathophysiology**. Current evidence from experimental model systems strongly highlights the neurovascular unit (NVU) as a relevant theatre of pathophysiology in MD. Within the framework of the neurovascular complex, coordinated physiologic mechanisms support regulation of cerebral hemodynamics at local (autoregulation and neurovascular coupling) and remote levels (coordinated vasoactivity and retrograde vasodilation) 211, 212. Within the framework of the NVU, a hub of interconnected pathological mechanisms exist which are linked to both AD and VaD. In a conceptual network pathophysiologic model of MD, oxidative stress, neuroinflammation, cerebral hypoperfusion, and BBB dysfunction operate synergistically to compromise local hemodynamic control mechanisms (e.g. dysautoregulation, neurovascular uncoupling) 213-219. Together, these pathophysiologic insults to the NVU and compromised hemodynamic control mechanisms create a cerebral microenvironment that is especially susceptible to neurodegeneration beyond the extent of either AD or VaD pathology in isolation. Vascular contributions to NVU dysfunction: Neurons are uniquely susceptible to acute or chronic hypoxia, which is introduced via cerebral hypoperfusion. Under the conditions of chronic hypoxia introduced in recovery following stroke or sCVD, arteriolosclerosis, or other vascular pathologies, the NVU operates in a condition where energy demand outpaces energy supply. Consequently, the NVU becomes a crucible of neuroinflammation and cellular oxidative stress which facilitates Aβ and tau aggregation and spread 139, 140, 220-222. Aβ and tau contribute to NVU dysfunction: Cerebral amyloid angiopathy enhances blood brain permeability, which triggers neuroinflammation and oxidative stress through multiple mechanisms and both CAA and tau disrupt neurovascular coupling 223, 224. Glial contributions to NVU dysfunction: (A) Under pathological conditions, microglia directly participate in BBB dysfunction by phagocytosing astrocytes and by releasing matrix metalloproteinases that degrade the basement membrane surrounding parenchymal arterioles 225, 226; and (B) In neuroinflammation, cytokine signaling triggers inducible nitric oxide synthase (iNOS) in astrocytes—potentially generating nitric oxide concentrations that are orders of magnitude higher than in physiologic conditions which may compromise hemodynamic control 218. Declining cholinergic system contributes to NVU dysfunction: (C) In AD, the basal forebrain cholinergic system undergoes early degeneration. Cholinergic innervation occurs broadly through the cortex and cholinergic neurotransmission plays a significant role in functional connectivity, neuroimmunomodulation, vasodilation, and glymphatic clearance 212, 227. Chronic and progressive diseases contribute to NVU dysfunction: Chronic diseases such as hypertension, hyperlipidemia, and diabetes contribute to remodeling the cerebral vasculature (e.g. cerebral arteriolosclerosis) and promote AD and VaD pathogenesis 228, 229. Furthermore, these chronic diseases contribute etiologically to cardiovascular disease which may then in turn, influence AD or VaD. Interestingly, evidence suggests that both angiotensin II signaling and insulin resistance promote neurovascular uncoupling 230, 231. Lastly, it should also be appreciated that AD pathology can directly contribute to CVD by facilitating autonomic dysfunction 232, 233. *Created in BioRender. Wohlfeld, C. (2025) https://BioRender.com/phke7ol*.

MD represents a distinct neuropathological entity characterized by the confluence of both AD and VaD pathologies, resulting in a clinical syndrome with accelerated progression and unique mechanistic interactions. It is not simply the additive burden of Aβ plaques, NFTs, and cerebrovascular dysfunction. Instead, the bidirectional synergism between these pathologies accelerates neurodegeneration more aggressively than either disease alone. **Figure 2** encapsulates the key mechanisms in AD and VaD and their interactions, elaborating the intricate pathophysiology of MD. For example, vascular pathologies impair cerebral Aβ clearance mechanisms mediated by perivascular drainage and glymphatic flow in the neurovascular unit. This reduction in clearance potentiates Aβ accumulation, which in turn exacerbates vascular dysfunction by promoting endothelial inflammation, pericyte loss, and vasoconstriction 72, 73. This then creates a self-reinforcing cycle of injury that is seen in MD. The pathological synergy is also seen in tau-mediated neurodegeneration; cerebral hypoperfusion induces hypoxia and metabolic stress, activating kinases that hyperphosphorylate tau and accelerate NFT development 74. Cerebral hypoperfusion also affects Aβ-driven neuroinflammation by amplifying microglial activation and cytokine release. The neuroinflammatory mechanism further damages the vasculature of the brain and promotes microhemorrhages or ischemic lesions 28.

While pure AD or VaD pathologies individually require substantial accumulation to manifest clinically, their synergy in MD creates an immense burden that exhausts neuronal resilience. This pathology creates a feedforward cycle of neurodegeneration, depleting synaptic reserves and reducing cognitive reserve more rapidly than either pathology alone 75. The intersection of AD and VaD mechanisms in MD fundamentally disrupts neurobiological homeostasis, thereby lowering the threshold for the clinical manifestation of dementia and contributing to more severe cognitive impairment.

### 4.2. Clinical manifestations of MD

MD presents a wide range of symptoms reflecting the combination of neurodegenerative and vascular pathologies. The clinical presentation includes features typical of AD or VaD, making it more complex and variable than either condition alone 2. Memory loss, often associated with AD, is one of the most prominent symptoms, while executive dysfunction, a hallmark of VaD, is equally significant in MD. In addition, patients frequently display motor symptoms such as gait disturbances and motor slowing, which are seen more often in VaD. Mood and behavioral changes, including depression, apathy, and agitation, are also common in MD 2. The multifaceted clinical presentations in MD more severely impair patients' daily functioning and quality of life. Understanding the range of symptoms helps to guide diagnosis and management strategies, as each symptom may require targeted intervention.

#### 4.2.1. Memory loss

Impairment of memory is one of the hallmark features of MD, similar to the progressive memory loss seen in AD. In AD pathology, the accumulation of Aβ plaques and tau tangles first affects the hippocampus and associated temporal lobe regions which are important for memory formation and recall 11. As a result, patients with MD often experience significant short-term memory deficits, such as forgetting recent conversations or misplacing items. Over time, these memory issues develop into long-term memory impairments, causing patients to forget important life events or familiar faces 5. However, the presence of vascular pathology can worsen memory loss. Chronic ischemia from SVD or strokes contributes to additional neuronal damage, further disrupting the brain's memory networks 60. This dual impact on memory systems in MD leads to more rapidly progressive and more severe memory loss than in cases of pure AD 46.

#### 4.2.2. Executive dysfunction

Executive dysfunction is also a common symptom in MD. Executive functions such as planning, organizing, problem-solving, and multi-tasking are essential for managing daily tasks and making decisions 76. Vascular lesions in the frontal lobes or subcortical areas, which are frequently affected in VaD, play a significant role in impairing these higher-order cognitive processes 76. Patients may struggle with tasks that require foresight and planning, such as managing finances, adhering to a medication schedule, or organizing household activities. Executive dysfunction can also manifest as a reduced ability to adapt to new information or changing circumstances, known as psychomotor slowing. Individuals' movements and thinking become slower, causing them to perseverate on specific thoughts or tasks, unable to shift focus or adjust to new demands, a symptom that often correlates with damage to the brain's white matter tracts. Executive dysfunction in MD often leads to reduced independence and increased reliance on caregivers 76.

#### 4.2.3. Motor symptoms

Motor symptoms are more pronounced in MD than in AD alone, due to the contribution of vascular pathology, which often affects brain regions involved in movement, such as the basal ganglia, cerebellum, and white matter tracts. One of the most common motor symptoms in MD is gait disturbance; patients may have difficulty initiating walking, exhibit shuffling steps, and display poor balance. The resulting increased risk of falls and injury significantly impacts their quality of life 60, 77.

#### 4.2.4. Mood and behavioral changes

Mood and behavioral changes are prevalent in MD, driven by the combined neurodegenerative and vascular pathologies. Depression, apathy, and agitation are particularly common. Patients may exhibit signs of sadness, withdrawal, and a general loss of interest in previously enjoyed activities. Depression can arise from the disruption of brain networks particularly with those affected by vascular damage 78. Apathy is another common symptom that can manifest as decreased initiative or energy, with patients showing little interest in social interactions or self-care. Apathy often correlates with damage to the brain's frontal lobes and subcortical areas, which are critical for motivation and executive function 79. Agitation and aggression are additional behavior changes that may arise, especially in later stages of MD, and can be distressing for both the patients and caregivers.

### 4.3. Diagnosis of MD

Proper diagnosis is essential for effective management and treatment, as MD often requires addressing both neurodegenerative and vascular factors. Given the complexity of the disease, diagnosing MD relies on a multifaceted approach that includes clinical evaluation, neuroimaging, and the use of biomarkers to fully look at all aspects of the disease.

The initial step in diagnosing MD is a comprehensive clinical evaluation, which includes an assessment of cognitive function, medical history, and a thorough neurological examination 2. Cognitive testing is crucial for identifying differences that are seen in AD and VaD. For example, patients with AD often exhibit significant memory loss, particularly in the domain of short-term memory, while those with VaD more likely show deficits in executive function, such as problem-solving 60. Patients with a combination of these deficits may likely have MD.

Understanding the patients' medical history is an essential component of clinical evaluation. A history of cardiovascular risk factors, such as hypertension, diabetes, and cigarette smoking, or a prior stroke may indicate a vascular contribution to dementia. Moreover, a family history of AD or other neurodegenerative diseases can indicate a genetic predisposition to AD. Since both conditions can present similar symptoms, the clinician must be careful in identifying symptoms that align with either AD or VaD to assess the likelihood of MD 62.

A neurological examination helps assess other neurological deficits such as motor symptoms or reflex changes that might suggest vascular contributions. Signs such as gait disturbances, motor slowing, or focal neurological deficits would clearly indicate VaD.

Clinical evaluations, while informative, often need to be followed with additional testing, particularly neuroimaging. Neuroimaging plays a pivotal role in diagnosing MD by allowing clinicians to visualize structural and pathological changes in the brain. Magnetic resonance imaging (MRI) and computed tomography (CT) are the most used neuroimaging techniques in dementia diagnosis. In AD, MRI scans often reveal brain atrophy, particularly in the hippocampus and medial temporal lobes 80. In contrast, VaD is characterized by the presence of vascular lesions such as white matter hyperintensities, lacunar infarcts, or large cortical infarcts, which can be seen in MRI or CT scans 66. Neuroimaging studies have shown that patients with MD likely have both extensive white matter changes and cortical atrophy 77. Additionally, amyloid positron emission tomography (PET) scans can be used to visualize Aβ plaques helping to distinguish cases where amyloid pathology is present alongside vascular damage 81.

While considerable progress has been made to improve diagnostic accuracy, overlapping imaging features and limited accessibility remain a challenge. As neuroimaging continues to evolve, it will continue to play a key role in the diagnosis of MD. In addition, as described later in this section, biomarker development is also key to improving the diagnosis of MD.

### 4.4. Risk factors for MD

The risk factors for MD overlap significantly with those of AD and VaD, encompassing genetic predisposition, cardiovascular health, and lifestyle influences, among others. A deeper understanding of these risk factors is essential for uncovering the mechanisms underlying MD and for developing effective preventive and interventional strategies. Here, we focus on several common cardiovascular and lifestyle risk factors that contribute to both AD and VaD components of MD.

#### 4.4.1. Hypertension

Hypertension is a critical driver of MD, accelerating the pathogenesis of both AD and VaD. Chronic elevation in blood pressure induces cerebral arteriolosclerosis 46. The vascular damage due to hypertension leads to microinfarcts and white matter lesions, disrupts neuronal connectivity, and creates a harsh environment that induces the spread of Aβ and tau pathologies 65, 82. The constant stress of hypertensive activity compromises BBB integrity by degrading tight junction proteins, which in turn causes neurotoxic plasma proteins to infiltrate the brain parenchyma and trigger neuroinflammation 50. The BBB dysfunction in turn affects the glymphatic system's ability to clear Aβ from the brain, allowing buildup of plaques causing further neuronal damage 28.

Cerebral arteriolosclerosis causes hypoperfusion and amplifies neurodegeneration. Briefly, chronic cerebral hypoperfusion induces hypoxia, which upregulates BACE1 and γ-secretase activity, promoting the cleavage of APP into pathogenic Aβ42 83, 84. Reduced cerebral blood flow also decreases neprilysin activity, limiting the degradation of Aβ and thereby exacerbating amyloid accumulation in the brain 25. Additionally, oxidative stress driven by hypertension activates GSK-3β and cyclin-dependent kinase 5 (CDK5), both of which hyperphosphorylate tau and promote NFT formation 85.

#### 4.4.2. Diabetes and insulin resistance

While AD is sometimes referred to as “Type 3 diabetes” due to the substantial role of diabetes in its pathogenesis 86, 87, diabetes is a significant risk factor for both AD and VaD components of MD as hyperglycemia and insulin resistance contribute to both vascular damage and neurodegeneration 88. Chronic hyperglycemia and insulin resistance contribute to cerebrovascular damage through multiple mechanisms. Hyperglycemia accelerates atherosclerosis by inducing oxidative stress and advanced glycation end-product (AGE) accumulation. This creates pro-thrombotic conditions that increase the risk of cerebral infarction 89. Clinically, diabetic patients show 2- to 4-fold higher incidence of stroke incidence, which implicates an increased risk for MD 90.

Continuous hyperglycemia also triggers the thickening of capillary basement membranes (i.e. microangiopathy). This structural change impairs oxygen and nutrient absorption and induces chronic cerebral hypoperfusion 91. The progression of hypoperfusion and microangiopathy are key factors that are linked to vascular cognitive impairment that can lead to MD.

Diabetes can also induce pericyte apoptosis and tight junction degradation through VEGF dysregulation. This can cause plasma protein leakage into brain parenchyma, triggering neuroinflammation that subsequently leads to neuronal damage 92. Rodent models with diabetes have shown an increase of 25%-40% BBB permeability due to chronic hyperglycemia 92.

#### 4.4.3. Hyperlipidemia

Hyperlipidemia, a very common but modifiable cardiovascular risk factor, accelerates MD pathology by driving cerebrovascular injury, neuroinflammation, and neurodegeneration 93, 94. Hyperlipidemia leads to the retention of LDL particles in the arterial intima, where prolonged exposure to ROS drives the oxidation of LDL, forming oxidized LDL (oxLDL) 95. OxLDL causes vascular endothelial cell injury and induces a macrophage inflammatory response in the arterial wall, leading to arteriosclerosis. As a major driving force of arteriosclerosis—primarily in large and medium sized arteries—hyperlipidemia causes cerebrovascular injuries and chronic hypoperfusion 82, compromises cerebral blood flow, and impairs entry of oxygen and nutrients to the brain 96, leading to demyelination and neuronal death 97.

OxLDL can also damage cerebral small vessels and induce BBB disruption 98-100. OxLDL particles then cross the BBB and activate microglia in the brain, triggering pro-inflammatory cytokine release 98-100. This neuroinflammation promotes Aβ aggregation while impairing its clearance by suppressing LRP1 101, 102.

Cholesterol imbalance is also directly linked to Aβ production and tau pathology. Excess intraneuronal cholesterol upregulates BACE1 activity and enhances amyloidogenic processing 103, 104. The byproducts of cholesterol oxidation have also been shown to induce tau hyperphosphorylation by directly activating GSK-3β and disrupting mitochondrial function by increasing ROS production. Both of these mechanisms accelerate NFT growth disrupting synaptic formation and leading to neuronal death 105, 106.

#### 4.4.4. Lifestyle factors

Lifestyle factors, including physical activity, cigarette smoking, diet, sleep, and cognitive engagement play a pivotal role in influencing the risk of MD. A sedentary lifestyle increases risk of both AD and VaD. Physical inactivity contributes to obesity, diabetes, and cardiovascular disease, all of which are risk factors for vascular and neurodegenerative damage 107. Dietary habits also significantly affect dementia risk. Diets high in saturated fats, processed food, and refined sugars contribute to metabolic disorders, such as diabetes and hyperlipidemia, which in turn, increase the likelihood of developing VaD and MD 108, 109. Conversely, adherence to a Mediterranean diet, rich in fruits, vegetables, whole grains, and healthy fats, has been associated with a reduced risk of AD and VaD 110. The anti-inflammatory and antioxidant properties of this diet are thought to protect against both cerebrovascular and neurodegenerative damage. Smoking is a significant risk factor for dementia. It promotes oxidative stress, neuroinflammation, and endothelial dysfunction. Indeed, chronic smoking has been linked to accelerated Aβ accumulation, increased risk for atherosclerosis, impaired cerebral blood flow and ischemic injury 111, 112, and accelerated cognitive decline 113.

Cognitive engagement is a protective factor against dementia. Individuals who participate in stimulating activities, such as reading, problem-solving, and social interactions, tend to have a lower risk of developing dementia. These activities are believed to build cognitive reserve, which enhances the brain's ability to compensate for age-related changes and resists the effects of both vascular and neurodegenerative damage 114.

### 4.5. Genetic studies

Genetic research has also been instrumental in identifying gene variants or mutations that increase susceptibility to MD. New genetic markers that are possibly linked to cardiovascular diseases are being explored for their role in MD. For example, variants in the clusterin (CLU) gene, which is involved in cholesterol metabolism and Aβ clearance, have been associated with increased risk for both AD and vascular pathologies 115. Similarly, the PICALM gene, known for its involvement in synaptic function and amyloid processing, is being examined for its contribution to both AD and cerebrovascular diseases 116. These studies highlight the complex genetic landscape of MD and suggest that it may be driven by a combination of neurodegenerative and vascular genetic risks. These genetic vulnerabilities show the importance of personalized clinical approaches for dementia prevention and treatment.

### 4.6. Novel biomarkers

Accurate and early diagnosis of MD remains a challenge due to the overlapping symptoms of AD and VaD. As a result, there is growing interest in identifying novel biomarkers that may enable physicians to more precisely detect the presence of MD and differentiate it from other forms of dementia.

The use of CSF biomarkers has enhanced our ability to detect the simultaneous presence of Aβ, tau protein, and neurofilament light chain (NFL), which are associated with AD, alongside markers of vascular damage, such as homocysteine and C-reactive protein (CRP). These biomarkers provide new insights into the molecular underpinnings of MD, revealing that both AD- and VaD-related processes may begin earlier than clinical symptoms manifest 117.

Another area of focus is plasma biomarkers, such as plasma tau (p-tau181 or p-tau217) as less invasive alternatives to CSF testing 118. NFL levels in the blood indicate axonal injury and are increased in both AD and VaD implicating potential utility as a marker for MD 117. Plasma-based biomarkers for Aβ and tau are being used to provide clinicians with more accessible tools for early diagnosis and monitoring of disease progression 119.

Further, biomarkers of the olfactory bulb are being investigated to distinguish between AD and MD. Olfactory dysfunction is a common early symptom in AD and MD 120. Studying the olfactory bulb allows researchers to correlate molecular changes with sensory deficits, potentially linking them to broader cognitive decline. Research has shown widespread disruptions in the homeostasis of olfactory marker protein (OMP) in dementia, with some alterations potentially specific to MD or AD 121. When measured in blood, OMP may serve as a promising biomarker to distinguish AD from MD, offering a valuable tool for differential diagnosis.

### 4.7. Pharmacological interventions for MD

Therapeutic treatments for MD are primarily aimed at the symptomatic relief of cognitive decline seen in AD while also addressing vascular risk factors that contribute to cognitive impairment. While there is no single medication approved specifically for MD, several drugs used to treat AD, or cardiovascular diseases have shown to be effective to various extent in some patients.

#### 4.7.1. Cholinesterase inhibitors and memantine

Cholinesterase inhibitors, such as donepezil, rivastigmine, and galantamine, are widely prescribed for AD and are also often used in cases of MD. These drugs work by inhibiting the breakdown of acetylcholine, a neurotransmitter that is typically deficient in patients with AD, leading to improvements in memory, attention, and other cognitive functions. Studies have demonstrated that cholinesterase inhibitors may provide modest benefits in improving cognitive symptoms in patients with MD 122. Memantine, another commonly used drug for AD, is an NMDA receptor antagonist; it acts by regulating glutamate activity, which is essential for learning and memory but when excessive can contribute to neurodegeneration. Memantine can be used alone or in combination with cholinesterase inhibitors and has been shown to offer symptomatic relief in mild to moderate cases of VaD and AD, but there are no studies showing its efficacy in MD, although the symptom relief from the studies does suggest it could be a potential treatment for patients with MD 122.

#### 4.7.2. Antihypertensive medications

Given the vascular component of MD, managing vascular risk factors is a critical component of MD treatment. Antihypertensive medications, such as angiotensin-converting enzyme inhibitors, beta blockers, and calcium channel blockers, help reduce blood pressure and decrease the risk of cerebrovascular events, such as stroke 123. The Systolic Hypertension in Europe (Syst-Eur), a multicenter randomized controlled trial (RCT) conducted by the European Working Party on High Blood Pressure in the Elderly (EWPHE), showed a 50% decrease of dementia incidence during a 2-year period in patients using long-acting calcium-channel blocker nitrendipine 124. Antihypertensives are used not only as preventative measures but also as potential treatments for dementia. In a study, patients with VaD and MD who used antihypertensive treatments to reduce and control systolic blood pressure showed improvements in cognitive scoring 123.

#### 4.7.3. Statins

Statins, widely used for managing hyperlipidemia, have been explored for their potential neuroprotective effects in dementia. Evidence suggests that statins may reduce cognitive decline by mitigating vascular risk factors, lowering cholesterol levels, and reducing inflammation**,** offering potential benefits for AD, VaD, and MD.

A longitudinal registry-based cohort study by Petek *et al.* 125 found that statin use was associated with slower cognitive decline in patients with MD and AD over time, particularly among those with significant vascular contributions to their pathology. These findings highlight statins as a promising intervention, especially for patients where vascular dysfunction plays a key role. While further research is needed to clarify the broader impact of statins, their dual role in cardiovascular health and neuroprotection makes them a compelling option for managing MD.

#### 4.7.4. Other potential treatments for MD

Emerging therapeutic strategies for MD should increase the focus on disrupting the self-reinforcing cycle of cerebral hypoperfusion, amyloid accumulation, and neuroinflammation through targeted molecular and delivery-based interventions. HIF stabilizers are one potential strategy to break the cycle of cerebral hypoperfusion and amyloidogenesis in MD. Studies demonstrate that HIF-1α stabilization reduces BACE1 expression and Aβ production under hypoxic conditions while promoting angiogenesis 126.

During sleep, the glymphatic system is more active in clearing neurotoxic proteins, such as Aβ, from the brain. Restoring circadian glymphatic function is another potential direction for MD management 127. Melatonin administration has been shown to suppresses MMP-9 activity, thereby stabilizing BBB integrity 128. In an AD transgenic mouse model, chronic administration of melatonin was able to reduce Aβ accumulation and increase soluble Aβ in the lymph nodes 129. As MD reflects a convergence of impaired perfusion and compromised clearance, interventions targeting sleep and drainage pathways could play a central role in a multi-modal treatment approach in the future.

In addition, active clinical trials are investigating the potential benefit of therapeutic agents such as GLP-1 receptor agonists and monoclonal antibodies against Aβ in MD management.

## 5. Animal models used in MD research

**Figure 3:**
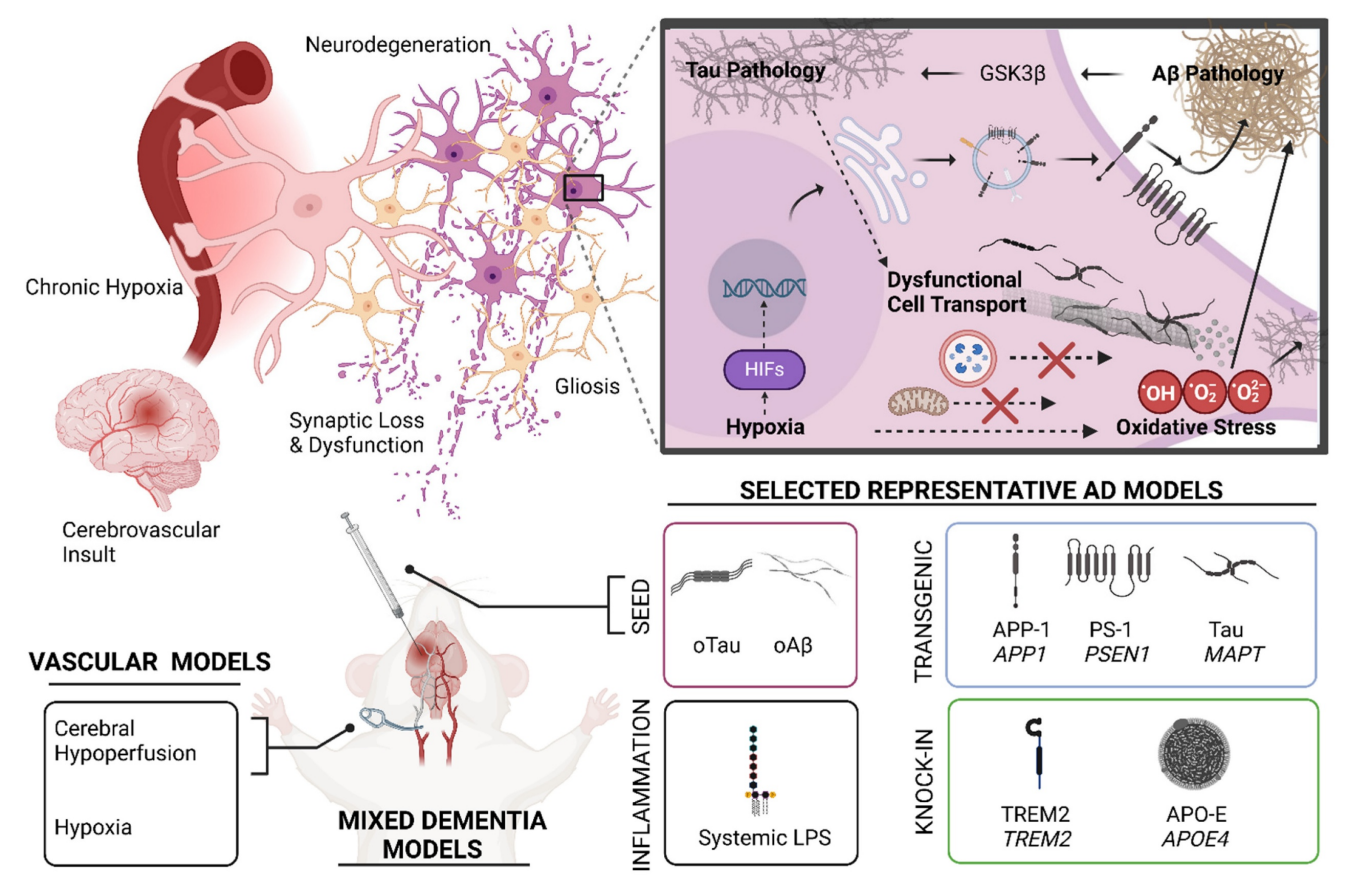
** Animal models for mixed dementia.** (Top Left) Pathophysiologic ramifications of chronic hypoxia introduced following a hypothetical stroke or other vascular pathology. Under the conditions of chronic hypoxia, various cellular and molecular features of Alzheimer's Disease pathophysiology are exacerbated and/or accelerated. (Top Right) Hypoxia introduces cellular oxidative stress—a redundant cellular pathomechanism in AD. Oxidative stress, tau pathology, and Aβ pathology are mutually interconnected via numerous mechanisms, as exemplified by glycogen synthase kinase 3β (GSK3β) 234 . Aβ pathology can likely initiate GSK3β-mediated phosphorylation of tau protein and mitochondrial proteins, which presumably promotes tau pathology, mitochondrial dysfunction, and oxidative stress 235. Additionally, acute and chronic hypoxia mediate transcriptional regulation via hypoxia inducible factors (HIFs). HIF-mediated transcriptional regulation likely contributes to beneficial adaptation to chronic hypoxia in the cerebral tissue microenvironment by promoting angiogenesis via upregulation of genes in the VEGF pathway. However, HIF-mediated transcriptional regulation may also be considered maladaptive, as HIFs also promote Aβ pathology by upregulating expression and activities of enzymes involved in amyloid precursor protein (APP) processing, introducing a bias towards production of pathologically relevant Aβ peptides such as Aβ_42_. (Bottom) Various mixed dementia models can be conceived by incorporating aspects of AD models with an appropriate experimental paradigm that introduces chronic hypoxia, such as a device that limits vascular patency or chronic exposure to hypoxic conditions. However, careful design must be exercised for such models, as different AD models showcase different aspects of AD pathology variably across the animal lifespan. Time course, background genetic environment in the model, and expression pattern of a pathologically relevant human transgene all influence selection of a model and interpretation of results. As an example, with very few exceptions, relevant tau pathology does not naturally present over the lifespan of rodents without use of a transgenic animal featuring a pathologically relevant MAPT variant (e.g. P301L, P301S), gene knock-in design, or seed injection of synthetic oligomeric tau (oTau) or patient-derived tau. Modeling Aβ pathology can be achieved using transgenic animals and/or gene knock-in of mutant or variant human genes implicated in the amyloidogenic pathway, such as amyloid precursor protein 1(*APP1*) or presenilin-1 (*PSEN1*). Additionally, synthetic oligomeric seed models (oAβ) and patient derived Aβ seeds have been successfully employed in animal models of AD. Other models include genetic knock-in of other pathologically relevant human genes that attempt to model other aspects of dementia such as neuroinflammation or Aβ clearance. These knock-in strategies usually are selected in an animal model that already produces tau or Aβ pathology, or both. Examples included are triggering receptor expressed on myeloid cells 2 (TREM2) KI and apolipoprotein E4 (APOE4) KI models. Systemic administration of lipopolysaccharide (LPS) may also be used to model neuroinflammation in an AD or MD model. Many other AD models could be used in a hypothetical mixed dementia model. Generally, an ideal animal model for MD would capture a pathophysiologic feature of AD that exhibits mechanistic synergy with a translationally relevant vascular pathological effect introduced as an independent variable which is counter-balanced by a control group in the experimental design. *Created in BioRender. Wohlfeld, C. (2025) https://BioRender.com/fen8ank*.

Animal models are indispensable in mechanistic studies and drug screening for MD. **Figure 3** shows the logistics and methods of the development and application of animal models for MD research. Many mouse models have been developed to model AD or VaD individually, but very few are available to model AD and VaD simultaneously. **Table 1** summarizes the key characteristics, advantages, and limitations of AD, VaD, and MD models, emphasizing how each model recapitulates aspects of neurodegeneration and vascular pathology.

### 5.1. Transgenic AD mouse models and their relevance in MD research

Transgenic rodent models are pivotal for studying AD due to their ability to replicate pathological aspects of human dementia via expression of human disease-associated genes. In most models, Aβ pathologies are modeled by introducing human AD-associated variants of APP, PSEN1, or both into mice. Because rodent wild-type tau protein does not normally form NFTs over the rodent lifespan, the use of mutated human tau, such as P301L or P301S, permits the introduction of human-like tau pathology in rodents. However, human MAPT P301L and P301S mutations are specifically associated with Frontotemporal dementia with parkinsonism linked to chromosome 17 (FTDP-17) and not broadly associated with dementia in AD populations. Nevertheless, it is considered that the pathological proteoforms that are produced in these transgenic tau models are still highly relevant to AD. An important limitation in modeling AD in rodents is that many models showcase Aβ pathology or tau pathology in isolation, whereas human AD patients exhibit these two simultaneously. As such, combined models featuring rodent expression of human transgenes relevant to both Aβ pathology and tau pathology have been created and are now commonly used. Important considerations for all transgenic models are the extent, cell-type, and neuroanatomical pattern of expression of transgenes and the genetic background of the rodent. As an example, the 5xFAD mouse model, which expresses human APP and PSEN1 transgenes with a total of five AD-linked mutations**—** the Swedish (K670N/M671L), Florida (I716V), and London (V717I) mutations in APP, and the M146L and L286V mutations in PSEN1, is significantly influenced by genetic background in the strain, with different strains presenting pathological features on considerably different time scales 130. Below we describe several common transgenic AD mouse models and their relevance in MD research.

#### 5.1.1. APP/PS1 transgenic mouse model

The APP/PS1 transgenic mouse models, including the 5XFAD model and others with one or more APP/PSEN1 transgenes, express human mutations in both APP and PSEN1 genes, and have been instrumental in advancing our understanding of AD. These mice develop amyloid plaques early in their life (i.e. ~ 6 months of age) 131. Recent studies have explored the role of vascular dysfunction in AD pathology in these mice, particularly in the context of MD. It has been shown that mice with APP/PS1 transgene mutations develop amyloid deposits in cerebral blood vessels as the mice age. The presence of Aβ plaques in the brain is associated with disrupted cerebral blood flow and impaired vascular function. The accumulation of amyloid plaques can lead to neuroinflammation and damage to blood vessels, resulting in reduced cerebral perfusion and contributing to cognitive decline 132. This confirms that vascular damage does in fact affect AD pathology and worsen symptoms. On the other hand, research has shown that induced vascular damage can worsen AD pathology, further showing the interplay between AD and VaD. Indeed, inducing vascular damage through chronic cerebral hypoperfusion (CCH) exacerbates cognitive decline in APP/PS1 mice 133. These findings underscore the critical role of vascular dysfunction in exacerbating amyloid pathology, suggesting that poor cerebral perfusion accelerates neurodegenerative processes associated with AD.

*In vitro* models using mouse neuroblastoma cells demonstrated that hypoxia stimulates the amyloidogenic pathway. This leads to increased Aβ aggregation 84. These *in vitro* findings were confirmed in studies using APP/PS1 AD mouse models. CCH induced by bilateral common carotid artery stenosis (BCAS) in APP/PS1 transgenic mice was shown to lead to HIF-1α-mediated BACE1 expression increase, exacerbating cerebral Aβ deposition and accelerating cognitive decline 83, 133. Other studies using PS1 mice showed that cerebral hypoperfusion also caused a decrease in neprilysin activation, impairing the clearance of Aβ and contributing to Aβ accumulation in the brain 134.

Another critical aspect of the APP/PS1 mouse model is its utility in studying the impact of Aβ on the BBB. Disruption of the BBB has been linked to both neuroinflammation and increased vulnerability to neurotoxic substances. Studies have shown that Aβ deposition in APP/PS1 mice leads to significant BBB disruption, resulting in increased levels of inflammatory mediators and further contributing to vascular pathology 135. The link between BBB integrity and cognitive impairment suggests that therapeutic strategies aimed at preserving BBB function could mitigate cognitive decline in patients with AD and MD. As such, by preventing or reversing BBB breakdown, it may be possible to disrupt the vicious cycle of inflammation and vascular damage, thereby slowing down the progression of MD.

#### 5.1.2. Mouse models for Tau pathology

Transgenic Tau mouse models overexpress mutant forms of tau protein, leading to the formation of NFTs and tauopathy 136. Mice expressing tau mutations such as P301L or P301S have been extensively used to investigate how tau aggregation leads to neuronal dysfunction, synaptic loss, and cognitive decline, recapitulating the tau pathology seen in AD patients 137. Tau transgenic mice begin to develop tau pathology relatively early, often showing signs of NFT accumulation and cognitive impairment by 6-9 months of age 138. These mice have proven invaluable for studying tau pathology independent of Aβ deposition. Like Aβ plaques, tau aggregation can also affect vascular health. Indeed, aged tau transgenic mice exhibit changes in their blood vessels, including spiraling morphology and reduced blood vessel diameter 85. *In vitro* studies have shown that hypoxia exacerbates tau hyperphosphorylation via HIF-1α-induced GSK-3β activation and suppression of phosphatase activity 139. *In vivo* studies using tau pathology mouse models confirmed these results; CCH increased tau hyperphosphorylation and reduced autophagy in these mice, leading to increased aggregation of NFTs and worsened neurodegeneration 140.

In addition to tau transgenic mouse models, the use of viruses has been valuable in understanding tau-related mechanisms in AD. Wegmann *et al.* 141 demonstrated that AAV-mediated expression of pathological tau in the mouse brain successfully mimicked key aspects of tau propagation observed in humans, including the misfolding of NFTs. Importantly, researchers observed that tau introduced to specific brain regions can spread to connected areas, showing tau's ability to propagate along neural pathways. This propagation model provides a controlled environment to study how tau spreads from one cell to another and how this spread correlates with neurodegeneration. The results validated AAV-based tau models as effective tools for replicating the prion-like transmission of tau pathology, making them valuable for future research on mechanisms driving tau-related neurodegeneration and for testing potential anti-tau therapeutics.

#### 5.1.3. 3xTg-AD mouse model

A common animal model for AD is the triple transgenic (3xTg-AD) mouse model. Developed by Oddo *et al.* 142, this model includes three human genetic mutations, APP Swedish, PSEN1 M146V, and tau P301L, that lead to the formation of both amyloid plaques and tau tangles. The 3xTg-AD mice is the only transgenic model to date that recapitulates both the Aβ and tau pathology seen in AD patients, making it a valuable tool for understanding the disease 142. Recent studies tracking pathology progression in 3xTg-AD mice have shown that Aβ deposits first emerge in the cortex by 6 months of age and gradually begin to accumulate in the hippocampus. By 12-15 months, Aβ is abundantly present in both the cortex and hippocampus 143. Interestingly, the opposite is seen with tau pathology. The hyperphosphorylation of tau begins in the hippocampus of the mice at 12 months of age and then proceeds to spread to the cortex with NFT structure becoming more apparent by 15 months of age 143. The sequential development of these pathological features provides researchers with a valuable window to investigate early disease mechanisms and potential therapeutic interventions.

Of particular relevance to MD research, the 3xTg-AD mice exhibit several features that may inform our understanding of disease comorbidities. Although primarily an AD model, these mice exhibit a robust neuroinflammatory profile—marked by pronounced astrogliosis and microglial activation—that may share common mechanisms with vascular contributions to cognitive impairment 144. Other studies have shown that BBB breakdown is an early marker of cognitive dysfunction, suggesting that the neuroinflammatory processes observed in 3xTg-AD mice may mirror those in human vascular cognitive impairment through disruption of BBB integrity 145.

Female 3xTg-AD mice have been shown to exhibit signs of cognitive decline earlier than males; deficits in spatial memory and learning are seen as early as 6-9 months of age. These sex differences are particularly evident in behavioral tests such as the Morris water maze, where females show impaired spatial navigation 146. In contrast, male 3xTg-AD mice typically do not display substantial cognitive impairment until 12 months or later, suggesting a slower progression of disease pathology. The accelerated cognitive decline in females correlates with earlier and more extensive Aβ accumulation, tau hyperphosphorylation, and neuroinflammatory markers, potentially driven by sex-specific factors such as the loss of estrogen's neuroprotective effects during aging 146. These findings highlight the importance of considering sex as a biological variable when using the 3xTg-AD model to study AD pathogenesis and develop potential therapeutic interventions.

### 5.2. Knock-in AD models

Knock-In (KI) models have been developed to complement transgenic models. In some cases, KI models attempt to surmount limitations of transgenic models. For example, KI models may permit more relevant gene expression patterns in rodent models of AD. Further, KI models can achieve cell-specific expression, delayed expression, or inactivation of transgene expression under certain conditions. Additionally, KI models have been used in synergy with transgenic models to explore interactions between Aβ or tau and other pathologically relevant genes, such as APOE or TREM2 147, 148. In this way, when used as complementary models to transgenic animal models, KI models permit deeper or more relevant investigation into specific pathological mechanistic details of dementia. A particularly relevant example is found in APOE KI models, with human APOE variant expression introduced in either 5xFAD or PS19 transgenic models 149, 150. Animal models employing this strategy have shown synergistic interactions between APOE4 and tau or Aβ pathology and demonstrate sex-specific effects in APOE3 KI mice, which is highly translationally relevant.

### 5.3. Other AD animal models

In addition to transgenic and KI models, there are a variety of other useful preclinical AD models that have been employed. Lipopolysaccharide (LPS) models, in which neuroinflammation is induced by LPS injection into the brain, are attractive given the central role of glia and neuroinflammation in AD 151. Lesion models, with targeted cell death in specific cell populations/neuroanatomical regions, have highlighted the importance of subcortical neuromodulatory cell populations in AD 152. Some of these neuronal populations, such as specific subpopulations of cholinergic neurons, experience early degeneration in AD, which has been proven pharmacologically and pathologically relevant 153. Various seed models have revealed important mechanistic insights into AD pathology. Seed models involve injection of artificial or patient-derived pathological proteoforms of Aβ, tau, or α-synuclein into targeted regions within the rodent brain. Seed models have shown prion-like transformation of endogenous wild-type rodent tau into pathological tau species following injection of a seed from human AD patients 154, 155. Furthermore, seed models routinely demonstrate spread within brain regions, across brain regions, and across hemispheres, which seem congruent to human cadaveric histopathology and PET neuroimaging studies in living human subjects with AD or MD.

Senescence models reveal relevant pathological mechanisms in animals that occur naturally over the aging process 156. This includes the Senescence Accelerated Mouse Prone 8 (SAMP8) model. SAMP8 mice are inbred AKR/J mouse strains that were recognized to have significantly shortened lifespan and multiple early senescence phenotypes as compared to control strains or other mice 157, 158. Importantly, SAMP8 mice exhibit spontaneously occurring Aβ and tau pathology and behavioral/cognitive deficits as compared to counterpart control strains 159-161. Displaying tau pathology without the expression of mutant human tau is an especially intriguing feature of the model. Beyond this, SAMP8 mice exhibit early enhanced oxidative stress, endoplasmic reticulum (ER) stress, impaired glucose metabolism, and neurodegeneration of cholinergic neurons, which arguably makes the model versatile and greatly enhances translational potential 162-164. Another strength of the SAMP8 model is that it can successfully mimic different stages of AD, unlike some models which deviate from the natural trajectory of human disease. Importantly, this may allow for screening putative interventions at earlier stages of the disease course.

Importantly, many of the models discussed above can be adapted by incorporating experimental design features that simulate aspects of vascular pathology, thereby enabling the development of MD models.

### 5.4. Animal models for VaD

Chronic cerebral hypoperfusion (CCH) models have become central to studying VaD due to their ability to mimic the gradual reductions in cerebral blood flow that contribute to neurodegeneration. CCH is commonly induced through bilateral carotid artery stenosis or occlusion in animals, simulating the progressive cerebrovascular insufficiency seen in humans with vascular cognitive impairment (VCI) 165, 166. The resulting chronic reduction in cerebral blood flow triggers several neuropathological alterations, such as white matter lesions, hippocampal atrophy, and synaptic dysfunction, all of which lead to cognitive impairments seen in VaD patients.

In a study by Zhou *et al.* 167, researchers investigated various animal models to determine the extent of cerebral hypoperfusion and whether any cognitive impairments are associated with it. They showed that mice that underwent bilateral carotid artery occlusion have 90% reduced cerebral blood flow (CBF) as early as 1-4 weeks after surgery, but demonstrated only acute deficits in spatial memory, which were shown in behavioral tasks such as the Morris water maze. Mice that underwent the bilateral common carotid artery stenosis surgeries showed two different results. Mice with 50% narrowing of the arteries showed a 20% CBF decrease, but no spatial memory deficits. On the other hand, mice with 75% narrowing of the arteries had a 30% decrease in CBF and showed significant spatial memory deficits. This suggests that during arterial occlusions, blood flow can be rerouted to maintain functionality; however, when arteries are chronically narrowed, significant cognitive impairments can occur. The cognitive impairments in these animals are associated with increased neuroinflammation and oxidative stress, suggesting that reduced CBF can cause white matter lesions and initiate neurodegenerative cascades involving inflammatory responses, highlighting the critical role that cerebrovascular pathology plays in cognitive decline 166.

## 6. The value of cell culture studies in MD research

Cell culture and co-culture models provide researchers with the unique opportunity to create controlled simplified experimental conditions, which can dissect the contributions of specific variables to the pathogenesis of AD, VaD, and MD. By manipulating certain variables, scientists can investigate the underlying mechanisms of disease progression, allowing for a more nuanced understanding of how AD and VaD components interact at the cellular level in MD pathogenesis. Furthermore, high-throughput drug screening enabled by these models accelerates the development of potential therapeutics.

### 6.1. Aβ and Tau pathologies revealed by cell culture studies

Cell culture models allow researchers to isolate and study specific factors contributing to AD and VaD pathology, such as Aβ production, tau phosphorylation, and endothelial dysfunction under controlled conditions. A central feature of AD pathology is the accumulation of Aβ peptides. *In vitro* models, such as neuronal cultures derived from human induced pluripotent stem cells (hiPSCs), have provided important insights into the mechanisms of Aβ production, aggregation, and toxicity. Cell culture systems using neurons derived from hiPSCs of patients with FAD replicate the genetic background of the disease, enabling researchers to observe how specific mutations influence cellular processes 168. Researchers can also examine Aβ oligomers to evaluate potential therapeutics to decrease aggregation. Kondo *et al.* 169 tested the effects of β-secretase (or BACE) inhibitors and antioxidants on Aβ plaques and found that BACE inhibitors reduced Aβ levels effectively, while antioxidants alleviated oxidative stress. However, some neurons responded well to BACE inhibitors but less effectively to antioxidants, or the other way around. This emphasizes the importance of personalized medicine, and how individual cellular profiles could significantly influence drug efficacy.

Tau hyperphosphorylation and the subsequent formation of NFTs are another hallmark of AD pathology. *In vitro* studies using both primary neuronal cultures and transfected neuronal cell lines have been instrumental in understanding the mechanisms of tau hyperphosphorylation and its role in promoting neurotoxicity 170, 171. Israel *et al.* 172 demonstrated that iPSC-derived neurons reprogrammed from fibroblasts of AD patients could replicate several disease features, including elevated levels of hyperphosphorylated tau protein 172. Moreover, *in vitro* models allow researchers to modulate tau phosphorylation directly by manipulating signaling pathways or genetic factors. Sotiropoulos *et al.* 173 investigated the functions of the tau protein *in vitro*, and found that it can form aggregates resembling those seen in neurodegenerative diseases, which may lead to neuronal dysfunction and cell death. These data suggest that tau not only helps stabilize microtubules but also influences other important cellular processes such as signaling and gene expression, thereby playing a more complex role than previously thought. These findings highlight the importance of studying tau in controlled settings to better understand its role in neurogenerative diseases and to advance the development of targeted therapies for tau-related dysfunction.

*In vitro* cell culture studies have also shown the interplay between vascular dysfunction and core AD pathology. For example, many studies have shown that hypoxia inhibits α-secretase activity while strongly stimulating the amyloidogenic β/γ-secretase pathway, resulting in accumulation of Aβ plaques 83, 84, 174.

### 6.2. Endothelial dysfunction in AD, VaD, and MD

Vascular contributions to cognitive impairment and dementia are characterized by cerebrovascular pathology including endothelial dysfunction, BBB breakdown, and cerebral hypoperfusion. Endothelial cells play a key role in maintaining the integrity of the BBB, and dysfunction in these cells is a major contributor to cognitive decline in VaD and AD 46. *In vitro* studies using brain microvascular endothelial cells (BMECs) are instrumental in understanding how vascular dysfunction contributes to neurodegeneration. A major challenge is the isolation of brain endothelial cells (BECs), as they constitute only 1-2% of the brain's total cell population. Further, isolated BECs are often contaminated with other cell types, compromising the purity and reliability of BEC cultures 73. To overcome this challenge, Daniels *et al.* 175 showed that immortalized human cerebral microvascular endothelial cells (iCMBECs) retain key properties of primary endothelial cells. iCMBECs have strong barrier integrity and maintain essential endothelial markers. When iCMBECs were exposed to pro-inflammatory cytokines, adhesion molecules were upregulated, facilitating the migration of immune cells such as monocytes and T cells across the endothelial layer.

BECs can also be used to test how hypoxic conditions induce vascular damage in the brain. In their 2012 study, Ogunshola and Al-Ahmad found that HIF-1 was activated when oxygen levels dropped, leading to changes in the endothelial cells (e.g. increased permeability). This increased permeability permits entry of harmful substances to the brain, consequently promoting inflammation and cell damage 176. Understanding HIF-1's influence on BBB function may help develop strategies to protect the brain during conditions like stroke or other hypoxic events.

Co-culture studies with endothelial cells and glial cells have been useful in understanding the BBB. Researchers have shown that interactions between endothelial cells and glial cells can lead to an increase in BBB permeability 177. During ischemia, signaling molecules released by glial cells increase the permeability of endothelial cells, allowing harmful substances to cross the BBB. Astrocytes help maintain the BBB structure and function by releasing factors that help to protect the BBB and promote endothelial cell survival. However, during hypoxia their protective functions may be compromised, contributing to BBB breakdown. Indeed, dynamic interactions between astrocytes and endothelial cells are essential for maintaining BBB integrity 178.

### 6.3. Glial cell dysfunction in AD and VaD

Glial cells, particularly microglia and astrocytes, play important roles in normal brain development and function. Cell culture studies have provided compelling evidence supporting a role for glial cell dysfunction in the pathogenesis of AD and VaD. Immortalized cell lines, such as microglia cell lines BV2 (mouse) and HMC3 (human) and astrocyte cell lines C8-D1A (mouse) and U373 (human), primary microglia and astrocytes isolated from neonatal mouse or rat brain, and human iPSC-derived microglia and astrocytes are all commonly used in cell culture studies. To mimic AD, these cells may be treated with Aβ oligomers, tau aggregates, or LPS. To model VaD, these cells can be subject to oxygen/glucose deprivation, hypoxia-reoxygenation, or hydrogen peroxide treatment. To evaluate their responses to these insults, parameters such as cell viability, activation status, cytokine release, ROS/NO production, and phagocytic capacity can be assessed. These cells can also be genetically modified through various approaches to assess how gene expression levels or specific variants/mutations influence their function or dysfunction in the context of AD or VaD.

Co-culturing microglia and/or astrocytes with neurons or brain microvascular endothelial cells enables the study of cell-cell interactions, either through direct physical contact or through the secretome (proteins, lipids, and microRNAs, etc.). It is noteworthy that Luchena *et al.* 179 generated a 2D triple co-culture model with murine astrocytes, neurons, and microglia through sequential seeding of each cell type. Using this approach, they revealed some important information regarding the interactions between these cell types in normal physiological conditions and in the context of AD. Co-cultures of human iPSC-derived neurons, astrocytes, and microglia are currently under active development for modeling neurodegenerative diseases 180.

### 6.4. High-throughput drug screening for MD treatment using cell culture models

High-throughput screening (HTS) and drug toxicity testing are critical components of drug discovery for MD. These processes rely heavily on cell culture models, which allow for the rapid identification of potential therapeutic compounds while also assessing their safety before moving to animal studies. In the case of AD and VaD, cell culture models are uniquely suited to facilitate the identification of drugs targeting specific disease pathways, thus enabling a more tailored approach to drug development. HTS has become an essential method for identifying potential treatments for AD and MD, particularly targeting the complex interplay between amyloid pathology and tau accumulation. For example, Gibbons *et al.* 181 used a seeded primary neuron model to screen small molecules for efficacy in modulating tau pathology. They identified specific compounds that effectively reduced tau phosphorylation and aggregation, highlighting their potential as therapeutic candidates for tauopathies associated with AD and potentially those with MD. Similarly, Hou *et al.* 182 demonstrated a novel high-throughput assay to monitor the proliferation of PC12 cells, which models neurodegenerative conditions, including AD. The results demonstrated that certain compounds significantly increased cell viability and reduced Aβ-induced toxicity.

HTS using VaD-relevant cell culture models has also yielded promising findings. A study by Tian *et al.* 183 investigated the pharmacological effects of Shenzhi Jiannao on VaD using *in vitro* approaches. Shenzhi Jiannao is a traditional Chinese medicine used to treat cognitive impairments, particularly those associated with VaD. It typically includes various herbal components believed to improve blood circulation, reduce inflammation, and enhance neuronal health 184. Their results indicated that Shenzhi Jiannao significantly enhanced neuronal viability and reduced apoptosis in cultured neurons exposed to hypoxic conditions. They also identified bioactive compounds within the prescription that modulated multiple signaling pathways, including those related to neuroinflammation and neuroprotection. These findings support the potential of Shenzhi Jiannao as a therapeutic agent for VaD and potentially the vascular components of MD.

## 7. Value of brain organoids in MD research

Traditional animal models and *in vitro* cell culture studies have facilitated our understanding of AD, VaD and MD, but they often do not fully capture the complexities of pathologies in the human brain. Brain organoids are emerging as promising tools for studying these conditions. These small, three-dimensional structures grown from stem cells mimic some features of the human brain. They are generated by taking stem cells, such as induced pluripotent stem cells (iPSCs), and differentiating them into various types of brain cells. Over time, these cells organize into tiny, self-assembling brain-like structures that can model parts of the human brain 185. For AD and VaD, brain organoids offer an important advantage as they allow researchers to study brain processes and disease mechanisms in ways that are more like human biology than available animal models. For example, human brain organoids have been used to study the buildup of Aβ and tau tangles. Moreover, brain organoids are useful for testing potential treatments, such as new drugs or gene therapies. While brain organoids hold immense potential for advancing our understanding of AD, VaD, and MD, they do have some limitations. For example, they still don't fully replicate the complexity of the human brain, and they may not reach full maturity, which can affect their ability to model disease processes faithfully 186.

### 7.1. Human specific pathways

One of the main challenges in studying AD, VaD, and MD using animal models is the lack of accurate representation of human-specific molecular and cellular pathways. Transgenic mouse models of AD, while informative, do not fully mimic the disease's human biology. For instance, the processing of APP and the subsequent formation of Aβ plaques differ significantly between mice and humans. Rodents typically do not develop the same extensive Aβ plaque deposition as seen in humans, and tau protein in mice does not form the NFTs typical of AD pathology in humans 187. As a result, key features of AD, such as Aβ aggregation and tau pathology, are only partially replicated in animal models, which limits their translational relevance. Human brain organoids overcome these limitations by enabling the study of human-specific molecular events and signaling pathways. For example, when brain organoids are derived from human iPSCs carrying familial AD mutations, they exhibit the progressive accumulation of Aβ and hyperphosphorylation of tau similar to that observed in the human brain 188. This makes organoids an invaluable model for investigating the early molecular mechanisms underlying AD pathology, including the abnormal processing of APP and tau hyperphosphorylation. These models also allow researchers to dissect the human-specific cellular responses to Aβ toxicity, such as the activation of microglia and astrocytes, which are involved in neuroinflammation and neuronal death 189.

Brain organoids also provide a novel platform for studying the effects of vascular risk factors such as hypertension, diabetes, and ischemia on brain cells. Brain organoids can closely mimic the complex cellular interactions and structures found in human brain tissue, making them particularly useful for understanding the pathophysiology of conditions like VaD that involve reduced blood flow and oxygen deprivation. Organoids placed in hypoxic conditions show cellular metabolic changes that cause increased neuronal cell death. Astrocytes respond by releasing pro-inflammatory cytokines, contributing to neuroinflammation 190. This highlights the value of brain organoids in revealing the molecular mechanisms of VaD and in guiding the development of targeted treatments.

### 7.2. Incorporation of vascular cells into brain organoids

Recent breakthroughs have enabled the development of vascularized brain organoids, by introducing endothelial cells and pericytes into the organoid structure 191. These vascularized organoids replicate the BBB, allowing scientists to study the effects of cerebrovascular dysfunction in neural tissue. This model incorporates various cell types, including neurons, astrocytes, and endothelial cells, which collectively reflect the brain's microenvironment. It has been reported that Aβ significantly disrupts the integrity of the BBB and impairs endothelial cell function, leading to increased permeability and neuroinflammation in vascularized brain organoids 192. These results highlight the relationship between amyloid pathology and vascular health, emphasizing that impaired vascular function can worsen neurodegenerative processes in AD. Vascularized human brain organoids are expected to play a significant role in mechanistic studies and drug development for MD.

### 7.3. Incorporation of microglia into brain organoids

With constant advancement in the field of brain organoids, researchers are increasingly focusing on the incorporation of non-neuronal cells such as microglia into organoids. Microglia are essential for homeostatic maintenance, immune surveillance, and neuroinflammatory signaling. Their integration into organoid systems shows promise in mimicking the environment of complex neurodegenerative conditions, where neuroinflammation plays a central role in disease progression.

Organoids are naturally able to develop microglia endogenously even in the absence of added mesodermal differentiation cues, although the number of microglia is very limited. These microglia-like cells exhibit canonical markers such as IBA1 and TMEM119. They demonstrate phagocytic capacity and respond to inflammatory stimuli, highlighting their functional relevance for studying innate immune processes 193.

To significantly increase the number of microglia in brain organoids, researchers incorporated iPSC-derived microglia into midbrain organoids. These exogenous microglia successfully integrated into the organoids and adopted diverse activation states. The presence of microglia led to enhanced neuronal maturation and increased electrophysiological activity 194. These findings support the notion that microglia are crucial for neuronal circuit formation and their absence may hinder the accuracy of later-stage neurodegeneration models. Organoids integrated with microglia were also able to respond to Aβ and tau, as well as secrete inflammatory markers, providing valuable evidence that this model can be used to study neuroimmune interactions in neurodegenerative disease 195. One study showed that cerebral organoids integrated with iPSC-derived microglia had the capacity to recapitulate microglial responses to Aβ and tau pathology, such as clustering around plaques and promoting neuroinflammation 196. These integrated microglia altered the trajectory of neurodegeneration and were instrumental in modeling the immune component of disease progression.

### 7.4. High-throughput drug screening using brain organoids

Brain organoids have been utilized in HTS. Park *et al.* 197 constructed a brain organoid model for use in drug screening. They developed a network-based drug-screening platform by integrating mathematical modeling and the pathological features of AD with human iPSC-derived brain organoids, including CRISPR-Cas9-edited isogenic lines. They used 1300 organoids from 11 participants to build a high-content screening system and test BBB-permeable FDA-approved drugs. This study demonstrated the potential of brain organoids in AD, VaD and MD drug development.

Others have refined HTS approaches for brain organoids by developing an automated pipeline for screening iPSC-derived cortical organoids, enabling rapid quantification of neuronal activity, viability, and synaptic integrity using calcium imaging and multi-electrode arrays (MEAs). This platform is particularly valuable for identifying neuroprotective compounds for the treatment of neurodegenerative diseases 198. Park and Mook-Jung 199 advanced the field by proposing a precision medicine framework using brain organoids, demonstrating their potential for personalized therapeutic screening. Their approach incorporated patient-specific genetic risk factors and multi-omics analysis to identify individualized treatment responses in neurodegenerative diseases. Du *et al.* developed a miniaturized 3D organoid platform that reduced media requirements to less than 2 μL per organoid while enabling ultra-HTS of more than 10,000 organoids per run 200. This standardized system may maintain reproducibility across screening batches, which addresses one of the main limiting factors in organoid research.

### 7.5. Limitations of brain organoids

Brain organoids provide a platform for studying complex human brain functions and mechanisms that cannot be fully replicated using *in vitro* cell culture systems or animal models. However, the use of brain organoids presents several limitations that researchers must navigate carefully. Brain organoids recapitulate the early development of the brain, while AD and VaD develop in the aging brain of patients 201. Researchers must be aware of this and use validation models of the observed changes of the brain organoids. Also, unlike animal models, organoids do not allow for behavioral or cognitive assessment. In addition, while brain organoids can mimic certain brain developmental aspects, they lack the complete array of cell types, structural organization, and functional connectivity found in a mature brain. Organoids can contain neurons, astrocytes, and other supporting cells, but they do not fully replicate the interactions between the cells found *in vivo* 202. Although brain organoids can be co-cultured with other cell types to model vascularization using endothelial cells, or microglia and astrocytes to model immune responses, it remains particularly challenging. Reproducibility is also a significant challenge in brain organoid research. Variability among organoids derived from the same stem cell source can result in inconsistencies in differentiation rates, cellular composition, and structural organization 202. Differences in culture conditions, researcher handling, microenvironmental factors, among others, can all cause differences in organoid development and potentially worsen these inconsistencies. As the field of organoid research continues to evolve, it is imperative to address these limitations to ensure reproducible and effective use of this promising technology.

## 8. Concluding remarks

Due to multiple factors, such as the aging population, the worldwide obesity/diabetes pandemic, advancements in diagnostic techniques, and increased clinical awareness, the prevalence and diagnosis of MD with coexistence of AD and VaD pathologies are on a steep rise. This urgently demands more research in understanding the complex pathophysiology of MD and calls for development of new drugs to target the pathways relevant to both AD and VaD. To this end, it is critical to develop *in vitro* and *in vivo* models to accurately simulate the human pathophysiology of MD and to provide platforms for drug screening. New animal models, novel cell culture and co-culture systems, and cutting-edge human iPSC-derived brain organoids, in combination with *in vitro* and *in vivo* genetic modifying technologies, multi-omics data acquisition/analysis, and artificial intelligence, present unique opportunities for MD research and drug development, enabling us to meet one of the most devastating medical and public health challenges of our time.

## Figures and Tables

**Table 1:** Commonly used animal models for mixed dementia preclinical studies

Animal Model	Description	Aβ Plaques	NFTs	Synaptic Defects	Advantages	Disadvantages	Relevance to MD	References
APP/PS1 Transgenic mouse models	Co-expression of human *APP* (Swedish, Florida, London mutations) and mutant *PSEN1* (M146L/L286V)	Yes (6 months)	No	Yes	Recapitulate early Aβ pathology; vascular amyloid mimics cerebral amyloid angiopathy	Limited tau pathology; vascular dysfunction secondary to Aβ.	Vascular amyloid (CAA) develops with aging; CCH exacerbates Aβ deposition via HIF-1α/BACE1 upregulation	131, 132, 133, 135
Tau Transgenic mouse models	Overexpression of mutant human tau (P301L or P301S); or AAV-mediated tau propagation	No	Yes (6-9 months)	Yes	Model NFT formation and prion-like spread; show vascular abnormalities	No concurrent Aβ pathology; tau mutations atypical for AD.	Induce vascular abnormalities (spiraling morphology); hypoxia accelerates tau hyperphosphorylation	85, 136, 137, 138
3xTg-AD mouse model	Triple mutations: *APP_Swedish_*, *PSEN1(*M146V), Tau (P301L)	Yes (6 months, cortex)	Yes (12 months, hippoca-mpus)	Yes	Only model with sequential Aβ and tau pathologies; mirrors human neuroinflammation	Delayed tau onset (12 months); pathology not fully concurrent.	Early BBB breakdown precedes cognitive decline; females show accelerated pathology. Vascular insults worsen tau spread	142, 143, 144, 145, 146.
APOE4 KI mouse models	Human *APOE* ε4 knock-in crossed with AD models	Yes	Yes	Yes	Model gene-environment interactions; high translational relevance	Complex breeding; variable phenotype penetrance.	Exacerbate CAA, microhemorrhages, and BBB disruption; accelerate Aβ/tau synergy	150, 147, 148, 149
SAMP8 mouse models	Inbred AKR/J strain with spontaneous aging pathologies (no transgenes)	Yes	Yes	Yes	No genetic manipulation; mimics aging-related metabolic/vascular dysfunctions	Non-AD-specific pathologies; slow progression.	Model aging-related oxidative stress/metabolic dysfunctions; vascular-hippocampal interactions mimic MD	156-164
CCH mouse Models	Surgical carotid artery stenosis (50-75% occlusion); reduces cerebral blood flow (CBF)	N/A	N/A	Yes	Induces VaD-like hypoperfusion; combinable with AD models	High mortality with severe stenosis; variable cognitive deficits.	75% stenosis reduces CBF by 30%, impairing Aβ clearance and inducing white matter lesions. Combined with AD models to study hypoperfusion-Aβ/tau synergy	134, 165-167
Seed Models	Injection of synthetic/patient-derived Aβ or tau seeds into brain regions	Induced	Induced	Yes	Flexible pathology induction; models protein aggregation	Acute injury confounds chronic pathology.	Hypoxia accelerates prion-like propagation of tau; models protein aggregation in vascularly compromised tissue	154, 155
LPS Models	Systemic LPS administration to induce neuroinflammation	No	Exacerb-ated	Yes	Rapid microglial activation; model immune-vascular crosstalk	Transient effects; non-specific inflammation.	LPS amplifies tau pathology via CDK5 activation in 3xTg-AD mice; models neuroinflammation-BBB disruption	151
